# Department of Surgery

**Published:** 1898-11

**Authors:** 

**Affiliations:** Secretary M’killip Veterinary College, Chicago


### DEPARTMENT OF SURGERY.

Edited by L. A. Merillat, V.S.,
SECRETARY M'KILLIP VETERINARY COLLEGE, CHICAGO.

Curative methods may be properly classified in the order of their importance into: 1, surgery; 2, sanitation, and 3, medical therapeutics. The veterinarian who is proficient in each may reasonably expect a successful career, while the one who disregards either or seeks only mediocrity in each is destined to a lower rank.

Therefore, the object of dedicating this department to the Journal and its readers is to bring one of these subjects, surgery, prominently and continuously before the readers of English veterinary literature; to encourage and urge a more frequent yet judicious application of the art of surgery as a curative measure, and give the reading veterinarian the benefit of the experience and deductions of others, and thereby improve and increase the domain of veterinary surgery.

The work of editing such a department is entered upon with a full knowledge of its magnitude and the responsibility such a duty entails, without, however, any claim of proficiency or special qualification for the work. The prime motive is more to add one small mite toward the advancement of veterinary surgery and induce others to exhaust, if possible, the other topics.

Modern surgery has supplanted the medical therapeutics of so many diseases and conditions that the veterinary practitioner is seeking greater skill in its application and higher attainments in the art generally; but since proficiency in surgery can only be acquired through practice, too much should not be expected from literary instruction. The object of this department will be accomplished if its readers are led to attain, more rapidly, the skill which their own self-training alone can achieve.

The successful surgeon is a cool-headed man of great resources, has a perfect knowledge of anatomy, is cleanly in his work, skilled in the use of instruments in hand, cautious if not proficient in diagnosis, and exercises the greatest care in securing and restraining his patients. He is both judicious and bold, and reluctant to undertake work simply for the purpose of performing a "beautiful operation" when the chances of success are limited. Such a procedure to him is excusable in the college clinic, but never in practice.

The methods of restraint are too well known to require any descriptive details. I might, however, mention that the practitioner should adopt particular methods and adhere strictly thereto, as a deviation from an adopted or accustomed method is too often a cause of accident. The twitch and single side line are sufficient for operations performed in the standing posture. If the head is pushed high and the animal prevented from going forward, accidents by this method are extremely rare. In the recumbent position, whatever method is adopted, the animal should be cast only upon a deep, soft bedding and tied taut, to prevent injury from struggling. Fractures of the pelvis and vertebræ—the usual accidents of this method—are thus prevented. The operating-table has its advantages, especially for unilateral operations, but since so many operations are bilateral, and so many require special positions of the body or limbs, the universal use of the operating-table cannot be recommended. The operating-room should be ventilated on all sides and kept aërated continually while not in use. The floor is bedded with pine-shavings to the depth of one foot. These are sprinkled daily with a solution of potassium permanganate. A clean rubber sheet or sterilized rug is spread over them at the seat of operation when the animal is cast and prepared for operation. If then the rules of aseptic surgery are carefully carried out pus will be a rare sequel in the ordinary operation.

*Anæsthetics.* The anæsthetics which will be found reliable and answering every purpose are:

(*a*) Chloroform for the horse and ox.

(*b*) Ether for the dog and cat.

(*c*) Cocaine (10 per cent.) for local anæsthesia.

(*d*) Ethyl-chloride spray for simple incisions.

A discussion of the relative merits of other anæsthetics which have come into use during recent years only leads to the conclusion that the above possess all the merit required of anæsthetics. Chloroform is particularly effective and safe in the horse, but dangerous in the dog and cat. In the latter ether is equally effective and safe. Cocaine, in a 10 per cent. aqueous solution, is a safe topical anæsthetic when administered in doses not to exceed 1.3 c.c. (20 m.).

The *administration of anæsthetics* will be discussed in the next issue.

